# Citizen scientist monitoring accurately reveals nutrient pollution dynamics in Lake Tanganyika coastal waters

**DOI:** 10.1007/s10661-022-10354-8

**Published:** 2022-08-19

**Authors:** Happiness A. Moshi, Ismael Kimirei, Daniel Shilla, Catherine O’Reilly, Bernhard Wehrli, Benedikt Ehrenfels, Steven Loiselle

**Affiliations:** 1 Tanzania Fisheries Research Institute, Kigoma Centre, P.O. Box 90, Kigoma, Tanzania; 2 Department of Aquatic Sciences and Fisheries Technology, University of Dar es Salaam, Dar es Salaam, Tanzania; 3 Tanzania Fisheries Research Institute, Dar es Salaam Headquarters, P.O. Box 9750, Dar es Salaam, Tanzania; 4 Department of Geography, Geology and the Environment, Illinois State University, Normal, IL USA; 5 Department Surface Waters – Research and Management, Eawag, Swiss Federal Institute of Aquatic Science and Technology, Kastanienbaum, Switzerland; 6 Institute of Biogeochemistry and Pollutant Dynamics, ETH Zurich, Zurich, Switzerland; 7 Dipartimento Biotecnologie, Chimica e Farmacia, University of Siena, INSTM, Via Aldo Moro 2, Siena, Italy; 8 Earthwatch Europe, 256 Banbury Road, Oxford, UK

**Keywords:** Citizen science, Lake Tanganyika, Nitrate, Phosphate, Turbidity

## Abstract

Several studies in Lake Tanganyika have effectively employed traditional methods to explore changes in water quality in open waters; however, coastal monitoring has been restricted and sporadic, relying on costly sample and analytical methods that require skilled technical staff. This study aims in validating citizen science water quality collected data (nitrate, phosphate and turbidity) with those collected and measured by professional scientists in the laboratory. A second objective of the study is to use citizen scientist data to identify the patterns of seasonal and spatial variations in nutrient conditions and forecast potential changes based on expected changes in population and climate (to 2050). The results showed that the concentrations of nitrate and phosphate measured by citizen scientists nearly matched those established by professional scientists, with overall accuracy of 91% and 74%, respectively. For total suspended solids measured by professional and turbidity measured by citizen scientists, results show that, using 14 NTU as a cut-off, citizen scientist measurements of Secchi tube depth to identify lake TSS below 7.0 mg/L showed an accuracy of 88%. In both laboratory and citizen scientist-based studies, all measured water quality variables were significantly higher during the wet season compared to the dry season. Climate factors were discovered to have a major impact on the likelihood of exceeding water quality restrictions in the next decades (2050), which could deteriorate lake conditions. Upscaling citizen science to more communities on the lake and other African Great Lakes would raise environmental awareness, inform management and mitigation activities, and aid long-term decision-making.

## Introduction

Freshwater ecosystems provide a wide range of ecosystem services (Dube et al., 2015) and yet are under increasing pressures linked to land use and demographic change as well as economic development (Lowe et al., 2019; Vörösmarty et al., 2010). Widespread human alterations of element cycles through improper wastewater treatment, unsustainable agriculture and insufficient management of storm-related pollution events have led to increases in nutrient pollution and a general eutrophication of a large percentage of lakes and smaller lentic ecosystems (Bogardi et al., 2012). Eutrophication of these key water resources poses a number of potential risks to human and aquatic life (Houser & Richardson, 2010; Morse & Wollheim, 2014).

The African Great Lakes are some of the world’s largest and deepest lakes, but are undergoing a number of challenges due to changes in their climate and catchments (Loiselle et al., 2014). This is compounded by the intermittent and limited monitoring across these transnational waterbodies (Chawira et al., 2013). Lake Tanganyika is a primary source of water for nearby villages, towns and cities, but the near-shore environment is also heavily used for fishing, shipping, agricultural activities, bathing, washing and transportation (Kelly et al., 2017; Kimirei & Mgaya, 2007). According to Spigel and Coulter (2019), the flushing time of the lake is about 7000 years and deep water has an age of ~300 years (Branchu & Bergonzini, 2004; Durisch‐Kaiser et al., 2011). Therefore, Lake Tanganyika is highly sensitive to pollution loads from both the surrounding catchment and atmospheric deposition (Gao et al., 2018; Langenberg et al., 2003; Yu et al., 2016, 2018). While having a comparatively low degree of land development, agriculture and animal husband ry are present in much of the catchment. Coastal areas, such as Kigoma, have an increasing population and partially treated or untreated wastewater posing major risks to coastal water quality where sediment and nutrient concentrations are significantly different than those of the open lake (Bergamino et al., 2007; Cózar et al., 2012). Coastal waters, however, present more complex optical conditions and require more in situ monitoring. These areas are also where the local population are directly impacted by changes in water quality (Shen et al., 2022).

Regular monitoring of the lake water quality is crucial to managing the lake environment (Plisnier et al., 2018, 2022).

Monitoring has successfully been done in open waters to explore changes in water quality (Azanga, 2016; Bergamino et al., 2010; Cohen et al., 2005; Gao et al., 2018; Kalacska et al., 2017; Karamage et al., 2016; Mziray et al., 2018). However, limited monitoring has been done along the shoreline, where major inputs occur (Moshi et al., 2022). Although remote sensing and laboratory techniques offer potential, these approaches are expensive and require trained technical staff for sampling and analysis. Moreover, there are spatial and temporal variations in nutrient input and their incorporation into the trophic web that reduce the effectiveness of regulatory seasonal spot monitoring (Desrosiers et al., 2013; Ehrenfels et al., 2021; Karube et al., 2010; Vermeulen et al., 2015). Likewise, remote sensing requires large in situ datasets for algorithm development and validation. There is, thus, a clear need for low-cost, locally determined methods that can complement data gathered by remote sensing and seasonal agency monitoring.

Citizen science is the involvement of non-scientist citizens in the gathering of scientific information and is based on a joint effort of professional scientists and members of the public, who can be involved in designing the scientific research, data collection, analysis and reporting of results (Cappa et al., 2018; Ceccaroni et al., 2017; Dickinson & Bonney, 2012; Eitzel et al., 2017). Citizen science has been widely used to monitor aquatic environments throughout the world (Hughes et al., 2014; Hyder et al., 2017; Loiselle et al., 2016; Thornhill et al., 2019). Studies in the northern hemisphere have reported the high standard of data acquired (Lévesque et al., 2017; Loperfido et al., 2010; McGoff et al., 2017; Muenich et al., 2016; Thornhill et al., 2017), but few validation studies have been performed in Africa. Spatial and temporal changes in turbidity, nitrate and water level in rivers and groundwater were successfully determined using citizen science (Rufino et al., 2018; Wand a et al., 2017; Weeser et al., 2018), but the feasibility of using this approach in African Great lakes has yet to be clearly demonstrated (Bishop et al., 2020). Understand ing the opportunities presented by citizen science to complement other monitoring approaches will also support locally based management strategies to mitigate nutrient pollution in these important waters. The objectives of the present study aim at supporting this agenda by first validating water quality data of nitrate, phosphate and turbidity collected by citizen scientists with those collected and measured in the laboratory by professional scientists. Second, the study aims at identifying the patterns of seasonal and spatial variations in nutrient conditions and forecast future scenarios based on expected changes in population and climate by 2050.

## Material and methods

### Study site and sampling design

**Fig. 1:**
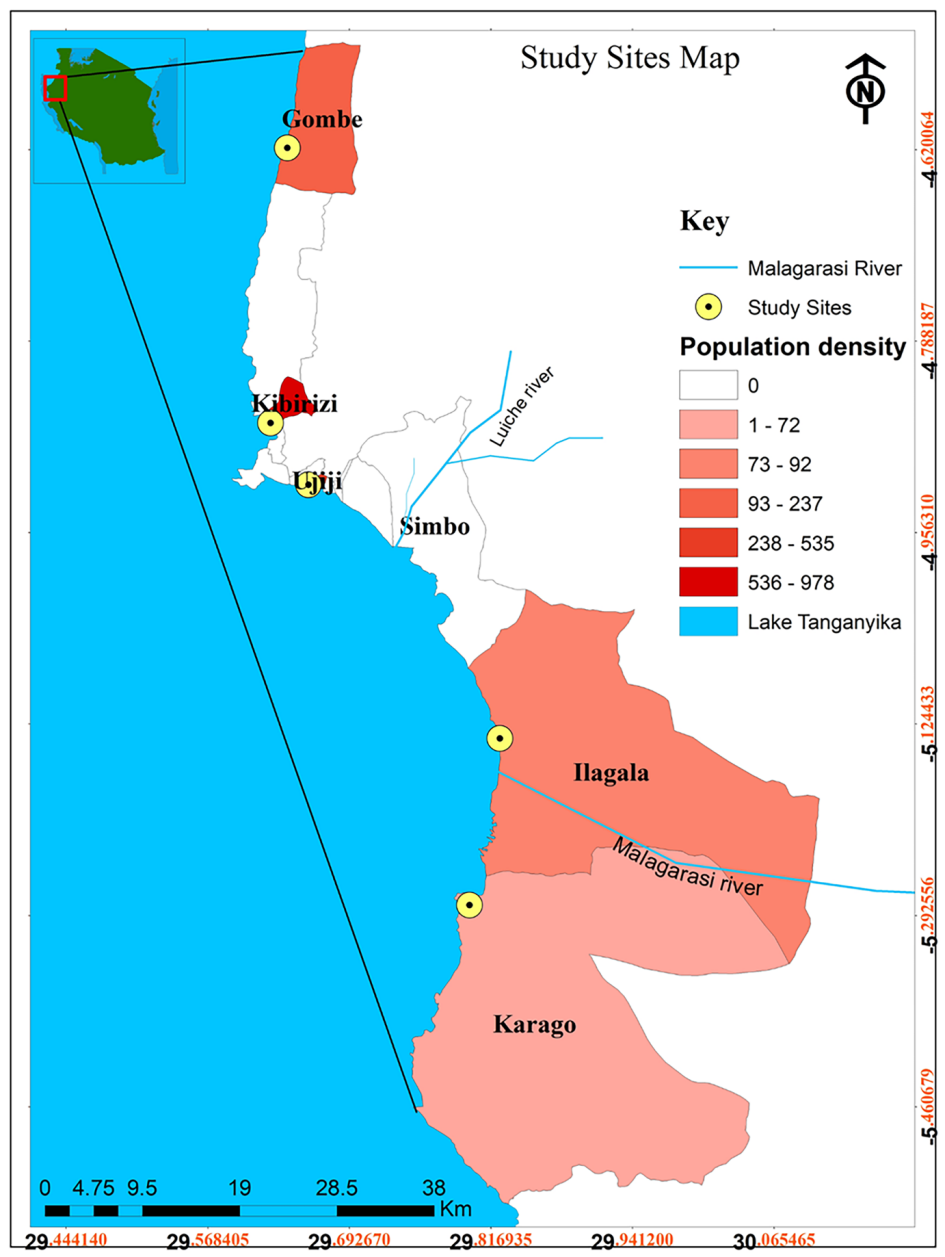
Map of northeeastern Lake Tanganyika showing the study sites

Water quality monitoring was conducted from May 2019 to April 2020 at 15 sites in five communities along the north-eastern coast of Lake Tanganyika, three sites in each of the following villages: Gombe, Kibirizi, Luiche Ujiji, Ilagala and Karago (Fig. 1). The sites were selected to cover differences in environmental characteristics (Table 1) including population density, land use, distance from the shore and from nearby rivers. Surface waters were sampled from a depth of 0.1–0.2 m using pre-rinsed plastic bottles attached to a 2-m pole to avoid the influence of the person and to avoid disturbing the lake sediment during sampling. A single sample was taken from the lake for each site and used by both citizen scientists and professional scientists for analysis.

**Table 1:** Location and description of study sites and associated sub-sites with their respective coordinates, distance from the shore (m), distance from the nearest river (m) and population

Site	Sub-site	Coordinates	Distance from shore (m)	Distance from nearest river (m)	Population	Description
Kibirizi	Kibirizi 1	-4.8611S, 29.6272E	6	15000	12,225	Peri-urban site located at 3–4 km from Kigoma town. Municipal discharge in the area (Fig. 1)
	Kibirizi 2	-4.8630S, 29.6286E	9	15000		
	Kibirizi 3	-4.8650S, 29.6233E	1200	15000		
Ujiji	Ujiji 1	-4.9244S, 29.6752E	7	3450	9040	Peri-urban site located 8–10 km from Kigoma town. The area is very close to Luiche river mouth and receives emissions from it. Farming activities near the lake shore take place (Fig. 1)
	Ujiji 2	-4.9180S, 29.6622E	15	4940		
	Ujiji 3	-4.9244S, 29.7063E	6	300		
Ilagala	Ilagala 1	-5.2119S, 29.8422E	4	160	21,246	Peri-urban site. This site is very close to Malagarasi river mouth and is impacted by its emissions. Farming activities take place (Fig. 1)
	Ilagala 2	-5.2116S, 29.8436E	13	78		
	Ilagala 3	-5.1552S, 29.8261E	6	6560		
Karago	Karago 1	-5.2813S, 29.7969E	12	9320	5456	Rural site in a closed bay and receives effluents from Malagarasi river (Fig. 1)
	Karago 2	-5.2877S, 29.7988E	22	9810		
	Karago 3	-5.2855S, 29.7894E	1700	10180		
Gombe	Gombe 1	-4.6269S, 29.5183E	4	15000	5270	Located within protected national park and surrounded by forest (Fig. 1)
	Gombe 2	-4.6344S, 29.6316E	5	15000		
	Gombe 3	-4.6411S, 29.6297E	10	15000		

### Recruitment and training of citizen scientists

A total of 250 individuals from five coastal villages were rand omly selected and included fishers, farmers, beach management units (BMUs), fish processors and fish sellers. Adult women and men (older than 18 y.o.a) filled out a structured questionnaire which was used to screen their willingness, environmental interest and availability to perform monitoring activities. A total of 150 out of 250 individuals (30 in each village) were recruited for training. All participants underwent a stand ard field training and safety course, which included theoretical and hand s-on experience on nutrients and turbidity measurements. For the theoretical session, training was conducted for two days (16 h), while the practical session was conducted over 5 days (40 h). During the theoretical classes, participants were taught about water quality issues, sources of nutrients pollution in the lake and implications on the services provided by the lake. During the practical session, participants, in groups of 5, practised sampling and analysis techniques using water samples provided for training (Fore et al., 2001). The participants were given time to practice using stand ard nutrient and turbidity kits under the supervision of the trainer, who was able to provide feedback.

Citizen scientists used the FreshWater Watch method to gather nutrients, turbidity and contextual information about the conditions of the site (Thornhill et al., 2018). Nitrate and phosphate measurements were taken colorimetrically in closed tubes using a specified volume (Kyoritsu Chemical-Check Lab, Corp., Tokyo, Japan). Phosphate concentrations were detected using an enzymatic technique (4-amino-antipyrine with phosphatase enzyme), and nitrate concentration estimation was based on the Griess method (Berti et al., 1988; Nelson et al., 1954). Citizen scientists compared the colour of the sample tube to a stand ard reference colour chart, assigning colour brightness to specific concentration intervals (Scott & Frost, 2017). Turbidity measurements were taken using stand ard calibrated Secchi tubes with detection limits of 14 and 240 Nephelometric Turbidity Units (NTU) (Preisendorfer, 1986).

Samples were taken at each site every month and divided into samples to be measured by both citizen scientists and those to be taken to the laboratory by professional scientists. Citizen scientists recorded measurements directly onto the FreshWater Watch app (iOS and And roid) or using the paper version. Results in hardcopy were transcribed onto the smartphone app by fellow citizen scientists to upload to the global database (https://freshwaterwatch.thewaterhub.org/).

Datasets were quality-controlled for consistency, internal and contextual, by professional scientists from Tanzania Fisheries Research Institute (TAFIRI) and Earthwatch directly from the online database. Internal consistency between data fields (water colour and turbidity) and comparisons between sampling locations and sampling events were used to identify transcription and methodological errors. Citizen scientists were contacted to correct or repeat any identified errors. A subset (1%) of reagent tubes (nitrate and phosphate) from each lot were checked in the laboratory using stand ard solutions.

### Laboratory measurements by professional scientists

Each sample was divided into three discrete water samples by professional scientists at the Tanzania Fisheries Research Institute (TAFIRI) for validation in the laboratory. The water samples were kept in small glass bottles (1 L) stored in cool boxes with ice and transported to TAFIRI Laboratory for analysis of nutrients, chlorophyll-*a* and total suspended solids (TSS) on the same day as the sampling.

In the laboratory, 50 ml of unfiltered water from the collected samples was used to analyse total nitrogen (TN) and total phosphorus (TP). The remaining water samples were filtered through glass microfiber filters (GF/C) for analysis of dissolved nitrates (NO_3_-N) and dissolved phosphate (PO_4_-P). TN, TP, NO_3_-N, and PO_4_-P concentrations were then analysed using a UV–Vis spectrometer (UV-2450PC, Shimadzu), following procedures described by (APHA, 1998). Laboratory nitrate and phosphate measurements were assigned to the same concentration intervals as used by the citizen scientist to allow for comparisons.

The GFC filters (0.45 µm diameter) for determination of total suspended solids were previously oven-dried at 103–105° C for one hour and weighed and recorded as initial weight (A, mg). After filtering each water sample, filters were dried again at 103–105° C and reweighed, recording their final weight (B, mg). The change in weight of the filter paper before filtration and after filtration of water sample was used to determine the amount of total suspended solids in mg/L, as

1 $$\mathrm{TSS }\left(\mathrm{mg}/\mathrm{L}\right)=\left(\mathrm{B}-\mathrm{A}\right)*\left.1000\right]/\mathrm{C}$$

where C denotes the volume of water filtered in litres (APHA, 1998).

The total suspended solids amount was used for validating citizen science turbidity measurements (Scott & Frost, 2017; Swift et al., 2006).

Chlorophyll-a was extracted with 90% (v/v) acetone after first disrupting the cells for 15 min by sonication. The samples were refrigerated (4 °C) overnight and re-sonicated the next day for 15 min before analysis using spectrophotometer.

### Data sources

Lake Tanganyika lies within the East African rift valley and is characterized by a four- to five-month cool (∼25 °C) dry season from May to September and a warm (∼28 °C) wet season from October to April (Savijärvi & Järvenoja, 2000; Verburga & Hecky, 2009). Kigoma receives a mean annual rainfall of about 935 mm and a monthly mean of 36.5 mm (Hunink et al., 2015). Strong south-easterly winds are prominent during the dry season, while the weaker winds from the northeast blows during the wet season (Docquier et al., 2016). Monthly precipitation, air temperature, wind speed and wind direction data for the whole study period (May 2019 to April 2020) were obtained from the Tanzania Meteorological Agency (Kigoma station), which is located approximately 7.2 km from the lakeshore.

To assess the influence of the anthropogenic land use on the nutrient load to Lake Tanganyika, basic land -use factors, i.e., the level of urbanization and agriculture, were qualitatively inferred from direct observations and Google maps. The population data for each study site were acquired from Tanzania National Bureau of Statistics 2012 population and housing census (NBS, 2013). According to the 2012 national census, the regional population of Kigoma is 2.1 million persons over an area of 45,000 km^2^. Higher population density characterises the lake shore areas (Fig. 1).

### Statistical analysis

Data were analysed using stand ard statistical methods, including paired t-Test (two paired sample for means), two-way analysis of variance (ANOVA), Pearson and Spearman correlations, to compare frequencies of concentrations and potential time-dependent drivers (R package × 64 4.0.2 and Realstats 2016). All data were tested with an alpha level of significance of 0.05 and using a Bonferroni correction for multiple correlations. Logistic regression models were used to calculate the probability of water quality variables to go over the limit across all the study sites and seasons (for the 2019/2020 and the prediction of the scenario in 2050). Training datasets (120) for the models were determined rand omly. The test datasets (60) were used to check for model accuracy. Water quality variables were assigned the binary value of 1 for concentrations above national limits, while 0 were assigned to measurements below the limit value. Limits were 0.10 mg/L NO_3_-N, 0.01 mg/L for PO_4_-P, 1.0 mg/L for TN, 0.1 mg/L for TP, 1 mg/L for TSS and 14NTU for turbidity. For comparisons between laboratory measurement of concentrations (continuous values) and citizen scientist recorded values (concentration categories), the central value of the concentration categories of the citizen scientist measurements was compared to laboratory concentrations converted into the same categories, using Cohen’s kappa. The projected change in monthly precipitation and populations for the time horizon 2050 followed estimates for an A2 scenario (Gebrechorkos et al., 2019) and a linear population increased based on past Tanzania census data (NBS, 2013). The estimated change in probability for concentration to supersede limits in 2050 was estimated using tested models and variables that were significant (p < 0.05) for the 2019/20 logistic regression models for each parameter (NO_3_-N, PO_4_-P, TN, TP, TSS, NTU).

## Results

### Validation of citizen science results

**Fig. 2:**
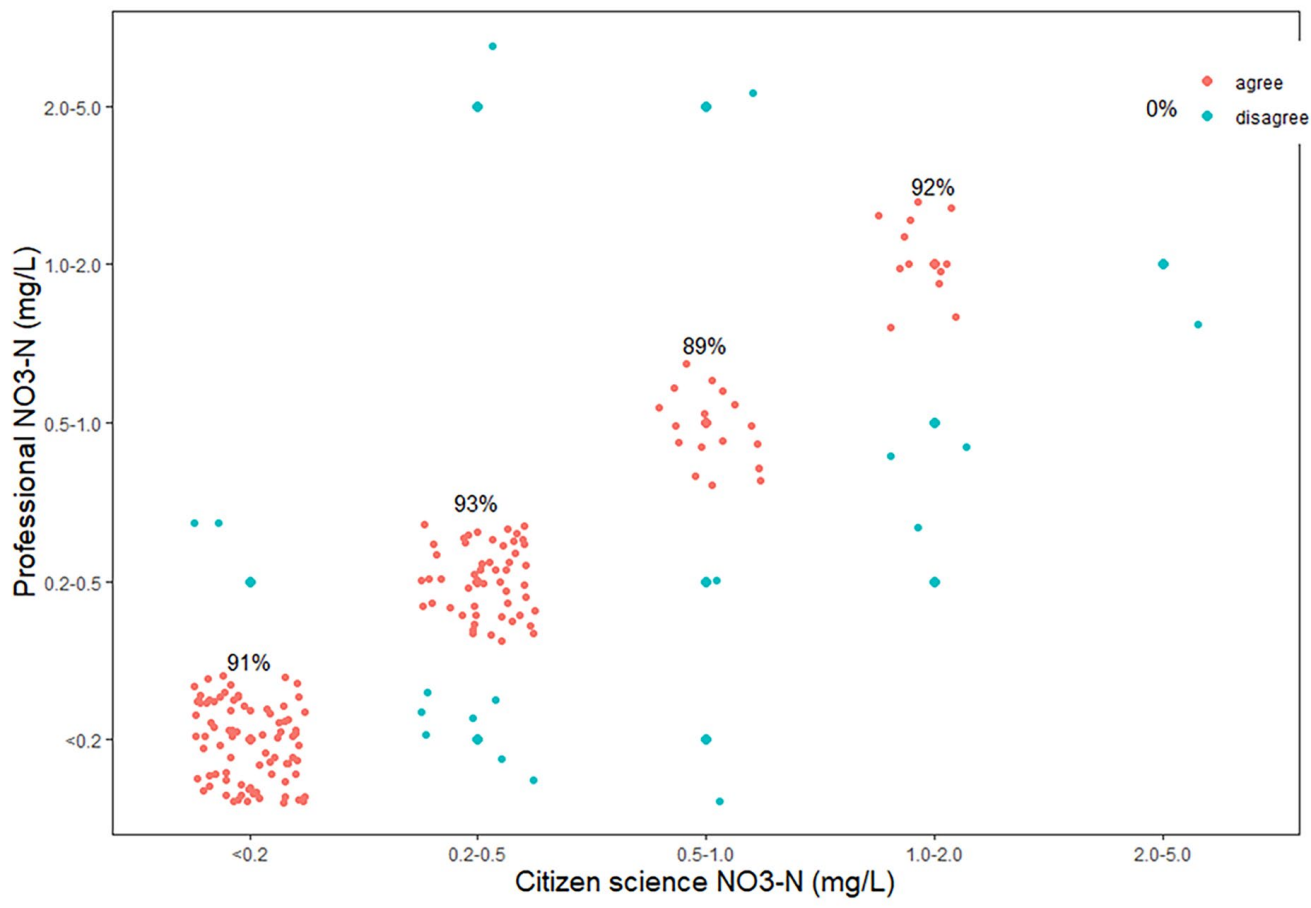
Correlation of nitrate concentrations measured by professional scientists versus those measured concurrently by citizen scientists with percent accuracy for each of the five concentration categories. Data points are randomly scattered around the category bins for better visibility

Nitrate concentrations measured by citizen scientists closely followed those determined by professional scientists with an overall accuracy of 91% (Fig. 2). There was an elevated and significant interrater agreement, with a Cohen’s kappa of 0.85 (p < 0.001). Of those measurements (9%) that did not agree, 7% were overestimates by the citizen scientists and 2% were underestimates. The majority (76%) of the incorrect estimates were within 1 concentration interval of the concentrations determined by the professional scientists. Differences between villages were observed, with Gombe having the highest accuracy (97%) and Karago with the lowest 81%, and the other three communities all with accuracy of 92% (Fig. 4). There was no significant difference between the accuracy in wet (91%) and dry seasons (89%).

**Fig. 3:**
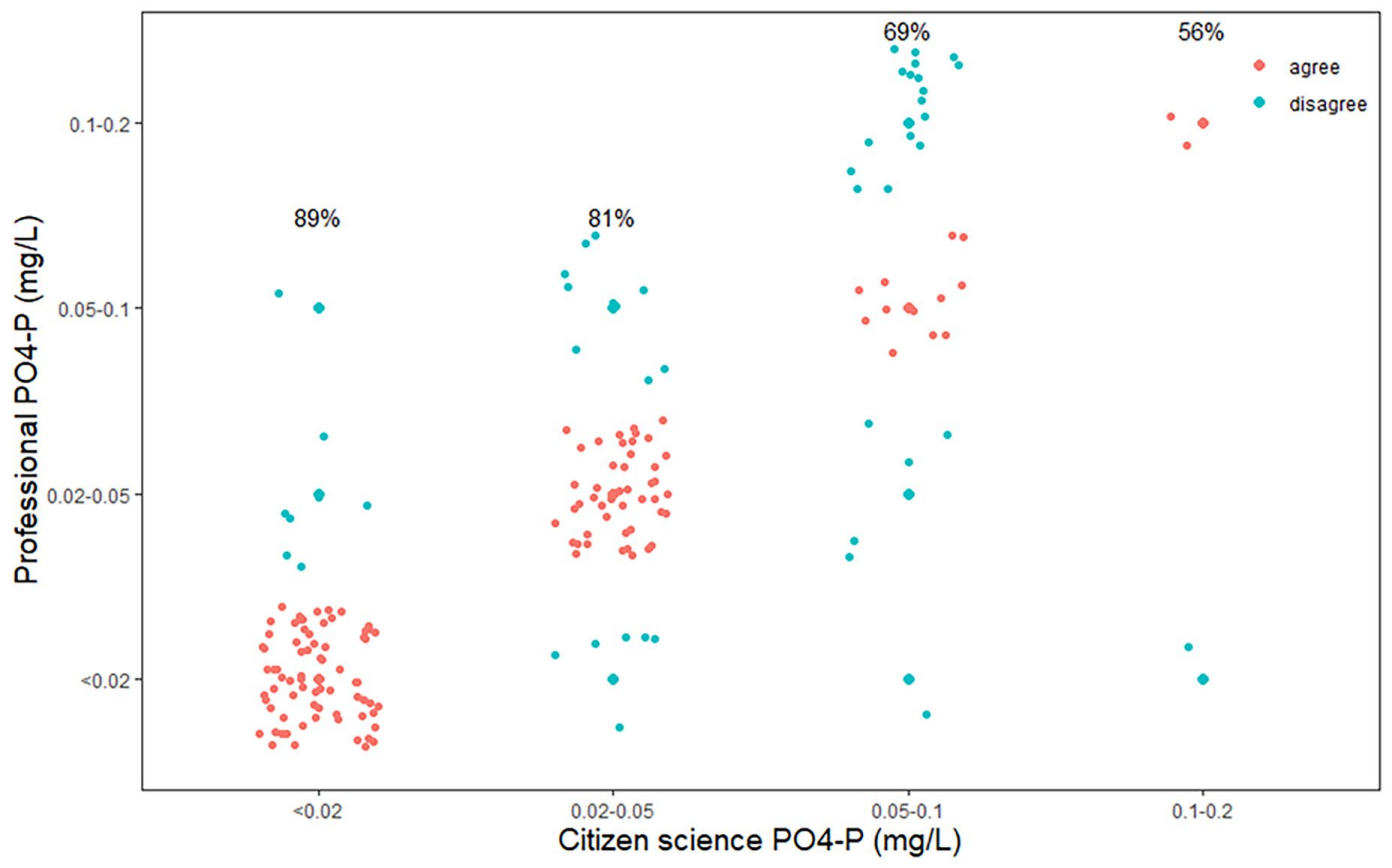
Correlation of phosphate concentration measured by professional and citizen scientists with percent accuracy for each concentration category. Data are randomly scattered in the concentration categories to improve visibility

**Fig. 4:**
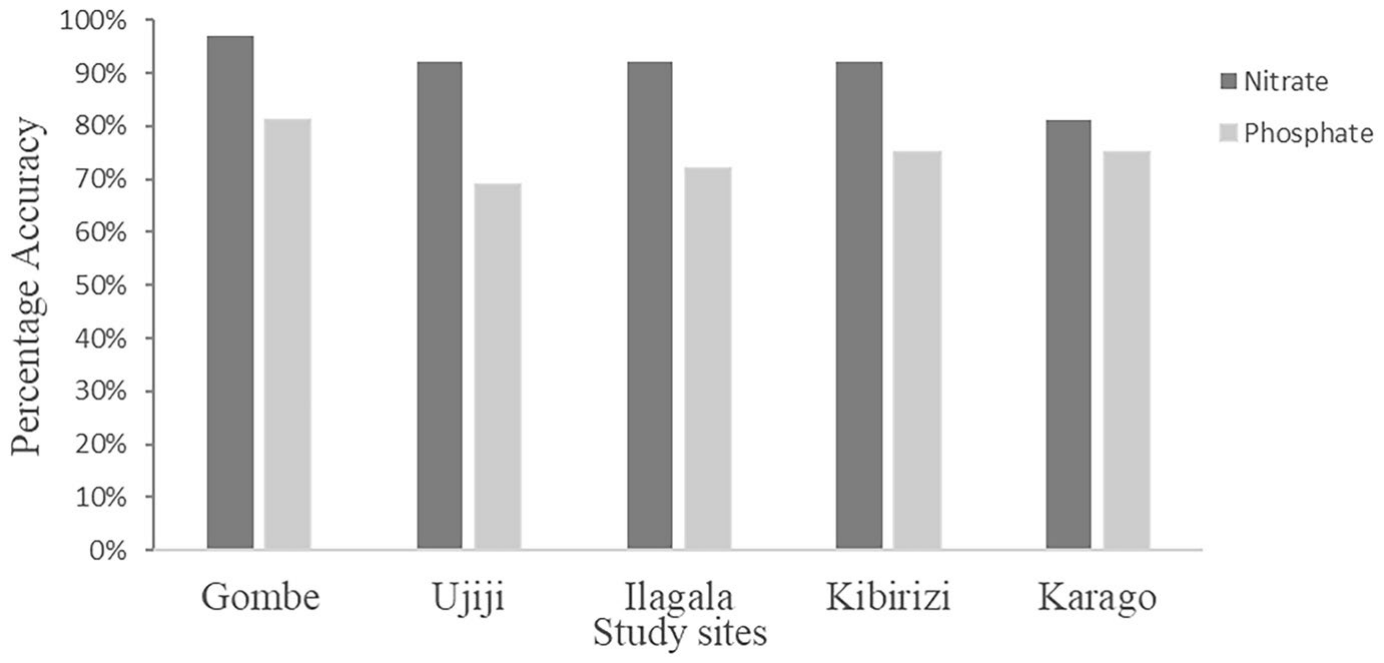
Percentage accuracy of nitrate and phosphate concentrations comparing professional and citizen scientists’ results across the study sites

Phosphate concentrations measured by citizen scientists also closely followed those determined by professional scientists but with a lower overall accuracy of 74% (Fig. 3). There was an elevated and significant interrater agreement, with a Cohen’s kappa of 0.61 (p < 0.001). Of those measurements that did not agree (i.e., 26%), 18% were overestimates by the citizen scientists and 8% were underestimates. Nearly all (96%) of the incorrect estimates were within 1 concentration interval of the concentrations determined by the professional scientists. Differences between villages were observed, with Gombe having the highest accuracy (81%) and Ujiji with the lowest 69%, while the other three communities ranged between 72 and 75% (Fig. 4). There was large difference between the accuracy in wet (66%) and dry seasons (87%).

The amount of particulate matter present in the lake was determined by multiple methods. A calibrated Secchi tube was used by citizen scientists to determine turbidity (NTU), while total suspended solids were determined in a laboratory by professional scientists. The Secchi tube has a minimum detection limit of 14 NTU and maximum detection limit of 240 NTU. From an initial analysis, the relationship between NTU and TSS was approximately 0.5 NTU for 1 mg/L. Using 14 NTU as a cut-off, citizen scientist measurements of Secchi tube depth to identify lake TSS below 7.0 mg/L showed an accuracy of 88%. Between sites, the highest accuracy was associated to Gombe (100%) and the lowest to Ilagala (70%), with the other villages between 86 and 96%. For measurements above 7.0, a strong linear relationship between NTU and TSS mg/L showed a correlation of 0.79 (n = 51, p < 0.001) and confirmed the relationship of 0.5 (0.51 ± 0.06) mg/L/NTU. The linear relationship contained measurements (n = 51) all five communities.

### Spatio-temporal dynamics of water quality variables

**Fig. 5:**
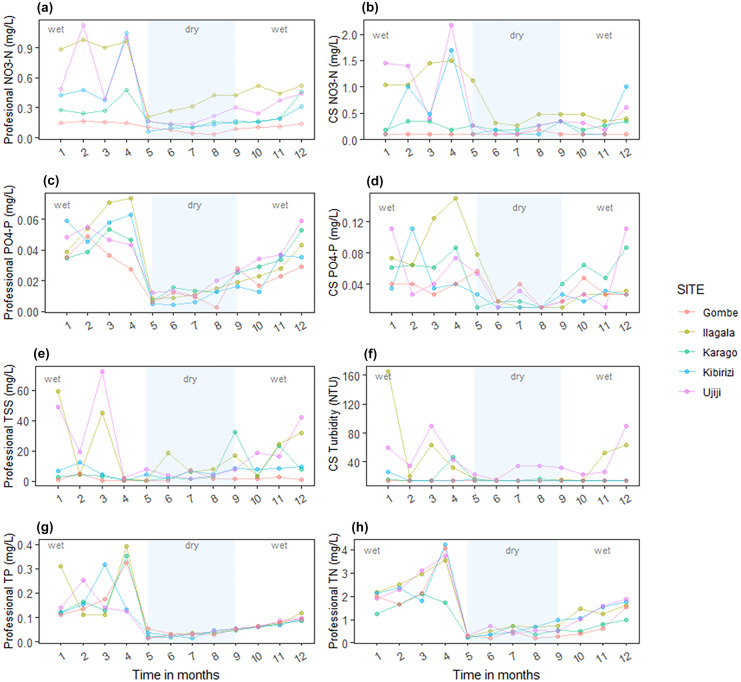
Temporal change in water quality at the five sampling sites in Lake Tanganyika. Professional refers to variable measured by professional scientists as displayed in (**a**), (**c**), (**e**), (**g**) and (**h**) and CS refers to variable measured by citizen scientists as shown in (**b**), (**d**) and (**f**)

For all parameters measured, there was a significant difference in concentration between wet and dry seasons (Tables 2 and 3). All measured water quality variables were significantly higher during the wet season compared to the dry season (Table 3), in both professional and citizen scientist-based measurements. Concentrations increased steadily at the beginning of the wet season (November and December) and reached their maximum in the second rainy season from March to April (Fig. 5). These seasonal dynamics were consistent in all villages except for Gombe, where concentrations of nutrients and particulates were generally lowest in both dry and wet seasons.

**Table 2:** Two-way analysis of variance of water quality values between sites and seasons and their interactions

Variable	Independent factor	DF	F	P
Dissolved nitrate	Sites	4,11	10.9	**< 0.001**
	Season	1,11	30.5	**< 0.001**
	Sites*Season	4, 11	1.9	0.131
Nitrate (citizen scientists)	Sites	4,11	5.3	**0.001**
	Season	1,11	9.1	**0.003**
	Sites*Season	4, 11	1.8	0.13541
Phosphates	Sites	4,11	1.6	0.188
	Season	1,11	90.6	**< 0.001**
	Sites*Season	4,11	0.9	0.44
Phosphate (citizen scientists)	Sites	4,11	1.2	0.313
	Season	1,11	18.5	**< 0.001**
	Sites*Season	4,11	1.0	0.399
Total nitrogen	Sites	4,11	1.5	0.218
	Season	1,11	57.3	**< 0.001**
	Sites*Season	4,11	0.7	0.616
Total phosphorus	Sites	4,11	0.2	0.955
	Season	1,11	30.8	**< 0.001**
	Sites*Season	4,11	0.1	0.969
Total suspended solids	Sites	4,11	4.8	**0.002**
	Season	1,11	6.0	**0.017**
	Sites*Season	4,11	2.6	**0.04**
Turbidity	Sites	4,11	5.6	**< 0.001**
	Season	1,11	7.3	**0.009**
	Sites*Season	4,11	2.4	0.057

Degrees of freedom (DF), F-values and p-values reported, significant p values are bolded

**Table 3:** Average water quality parameters measured by professionals in the laboratory and citizen scientists (CS, using central values of measured concentration ranges) during dry and wet season across the study sites, with ± standard deviations

**Dry Season**								
Site	Nitrate (mg N/L) Lab	Nitrate (mg N/L) CS	Phosphate (mg P/L) Lab	Phosphate (mg P/L) CS	Total nitrogen (mg N/L)	Total phosphorus (mg P/L)	Total suspended solids (mg/L)	Turbidity (NTU)
Karago	0.14 ± 0.02	0.25 ± 0.06	0.01 ± 0.006	0.02 ± 0.01	0.44 ± 0.17	0.03 ± 0.01	7.8 ± 12.2	14 ± 1.1
Ilagala	0.33 ± 0.09	0.53 ± 0.3	0.01 ± 0.004	0.02 ± 0.02	0.58 ± 0.17	0.03 ± 0.01	10.1 ± 6.8	14 ± 0.85
Kibirizi	0.11 ± 0.03	0.17 ± 0.09	0.009 ± 0.004	0.02 ± 0.008	0.54 ± 0.26	0.03 ± 0.01	4.9 ± 2.3	14 ± 0.8
Ujiji	0.19 ± 0.06	0.2 ± 0.1	0.01 ± 0.006	0.02 ± 0.02	0.48 ± 0.14	0.03 ± 0.01	4.8 ± 2.6	**28** ± **7.4**
Gombe	0.07 ± 0.02	0.1 ± 0.03	0.01 ± 0.008	0.03 ± 0.01	0.29 ± 0.1	0.03 ± 0.01	2.3 ± 2.6	14 ± 0

**Wet season**								
Karago	0.3 ± 0.11	0.27 ± 0.08	0.04 ± 0.009	0.067 ± 0.01	1.3 ± 0.52	0.14 ± 0.09	6.5 ± 7.1	19 ± 17
Ilagala	0.74 ± 0.21	0.89 ± 0.45	0.04 ± 0.02	0.07 ± 0.04	**2.21** ± **0.78**	0.17 ± 0.11	24 ± 21	**58** ± **47**
Kibirizi	0.43 ± 0.27	0.64 ± 0.57	0.04 ± 0.01	0.04 ± 0.03	**2.1** ± **0.9**	0.1 ± 0.08	7.1 ± 3.6	15 ± 4.2
Ujiji	0.58 ± 0.32	0.93 ± 0.67	0.05 ± 0.008	0.06 ± 0.04	**2.21** ± **0.84**	0.12 ± 0.06	**31** ± **22**	**52** ± **26**
Gombe	0.14 ± 0.02	0.1 ± 0	0.03 ± 0.009	0.03 ± 0.008	1.77 ± 1.11	0.14 ± 0.08	1.7 ± 1.5	14 ± 0
TBS ^a^	10–25		0.1–2.2		1.0 excluding nitrate	6	100	5–25
WHO ^b^	10		0.1		NA	1–4	25	5

The bolded values present measurements exceeding the TBS and WHO threshold values for drinking water

^a^Tanzania Bureau of Stand ards (TBS) (TBS, 2003)

^b^World Health Organization (WHO) (WHO, 2004)

Comparing villages, dissolved nitrates measured by both professional and citizen scientists were significantly different between sites (Table 2). The highest concentration of dissolved nitrate (mean ± SD = 0.46 ± 0.27 mg/L) was observed in Ilagala, whereas the lowest values were recorded in Gombe (mean ± SD = 0.10 ± 0.04 mg/L). Phosphate showed a similar spatial pattern, with concentrations as high as 0.03 ± 0.02 mg/L (mean ± S. D) in Ilagala and as low as 0.02 ± 0.01 (mean ± S.D) in Gombe, but the differences were not significant across sites. Total suspended solids and turbidity showed significance difference across sites (Table 2). Gombe reported the lowest concentrations of total suspended solids (mean ± SD = 1.9 ± 2.0 mg/L) and turbidity (less than 14 NTU), which both reached the maximum values in Ujiji with total suspended solids, mean ± SD = 20 ± 21 mg/L and turbidity = 42 ± 23 NTU (mean ± SD).

Putting these measurements into context, turbidity data measured by citizen scientists exceeded both Tanzania Bureau of Stand ards (TBS) (5–25 NTU) and World Health Organization (WHO) (5 NTU) permissible limits during the wet months in Ilagala and Ujiji and for Ujiji also during the dry months (Table 3). Total nitrogen was above TBS limits (1 mg/L excluding nitrate) in Ilagala, Kibirizi and Ujiji (Table 3). Nitrates, phosphates, total phosphorus and total suspended solids were below the permissible limits for drinking water in both seasons (Table 3).

### Drivers influencing nutrients and turbidity concentrations and future scenarios

For identifying the main influencing factors on the nutrient and turbidity concentrations, we applied logistic regression models with future climate forecast, hydrological and land -use drivers as explanatory variables. We furthermore used the models to estimate the probability for the water quality parameters to exceed the TBS and WHO concentration limits for two scenarios–the study period (2019/2020) and 2050. All the models were significant and provided similar accuracies with both the training and test datasets, from 73 to 95% area under the curve (AUC).

Climate factors were found to significantly influence the probability of exceeding water quality limits. Among those, precipitation explained most of the variations and was associated with an increased probability of elevated concentrations of dissolved nitrate, phosphate, total nitrogen, total phosphorus, and total suspended solids. Wind direction was strongly associated with elevated concentrations of phosphate, total nitrogen and total phosphorus (Table 4). Increasing wind speed had a negative impact on the probability of exceeding limits for phosphate, total nitrogen, total phosphorus and total suspended solids. Increased distance from local rivers was associated with lower turbidity in the lake, while increasing population was related to concentrations of total nitrogen above the accepted limits.

**Table 4:** Climate and geographic variables influencing the probability that water quality concentrations exceeded TBS and WHO concentration limits

	Logistic model p-values for different water quality variables with respect to climatic, geographical and population drivers					
Explanatory Factors	Nitrate	Phosphate	Total nitrogen	Total phosphorus	Total suspended solids	Turbidity
Wind direction	N.S	**0.0007**	**0.009**	**0.012**	N. S	N. S
		(0.2)	(0.27)	(0.15)		
Wind speed	N.S	**0.008**	**0.001**	**0.0001**	**0.003**	N. S
		(-2.45)	(-4.21)	(-2.42)	(-1.19)	
Rain	**0.0002**	**1.3E-06**	**0.002**	**0.009**	**0.003**	N. S
	(0.03)	(0.04)	(0.1)	(0.04)	(0.01)	
Distance from the river	**0.002**	**0.002**	N. S	N. S	**0.003**	**1.29E-05**
	(-0.0002)	(-0.0002)			(-0.0001)	(-0.0002)
Population	N. S	N. S	**0.007**	N. S	N. S	N. S
			(0.0006)			
Intercept	2.96	-8.24	-26.58	-10.42	7.12	0.08
Model Accuracy	0.95	0.94	0.92	0.74	0.92	0.87

The p-values are bolded for significant variables relative to each concentration limit with N.S representing non-significance. Model coefficients shown in parenthesis below each significant p-value

**Fig. 6:**
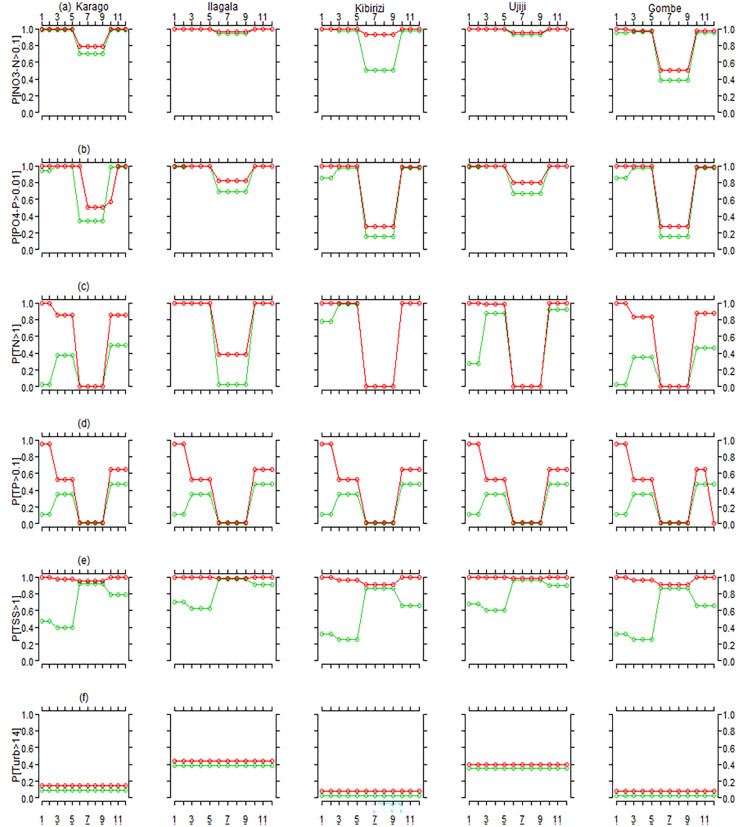
Probability of water quality parameters to go over the defined limit throughout the year across study sites during the study period of May 2019 to April 2020 (green line) and 2050 (red line). **a** probability of nitrate to go over 0.1 mg/L (P[NO3-N > 0.1], **b** probability of phosphate to go over the limit of 0.01 mg/L (P[PO4-P > 0.01], **c** probability of TN to go over the limit of 1 mg/L (P[TN > 1]), **d** probability of TP to go over the limit of 0.1 mg/L (P[TP > 0.1]), **e** probability of TSS to exceed 1 mg/L (P[ TSS > 1]) and **f** probability of turbidity to surpass 14 NTU (P[ Turb > 14])

In line with precipitation being the major driver, there was an elevated probability that nutrient and particulate conditions would exceed accepted limits in the rainy season. During the study period (2019–2020), the probability for nitrate, phosphate, turbidity, TSS, TN and TP ranged from 0.94 to 099, 0.85 to 0.99, 0.028 to 0.38, 0.25 to 0.90, 0.028 to 0.99 and 0.11 to 0.47, respectively, across sites in the rainy season and from 0.38 to 0.99, 0.16 to 0.99, 0.028 to 0.38, 0.028 to 0.38, < 0.01 to 0.99, and 0.6 to 0.35 for nitrate, phosphate, turbidity, TSS, TN and TP, respectively, in the dry season (Fig. 6). The analysis furthermore reveals that the probability of exceeding acceptable limits was highest for the dissolved nutrients, with 0.91 and 0.78 for nitrate and phosphate, respectively.

Compared to our study period, the probability for exceeding WHO and TBS limits was higher in the future 2050 scenario. TSS, nitrate and phosphate were most affected in the future scenario with higher probability of 0.97, 0.91 and 0.84, respectively, of surpassing the limits (Fig. 6).

## Discussion

### Citizen science for monitoring African Great Lakes

Coastal communities in many Great Lake countries have been involved in monitoring local fisheries, through beach management units (Bulengela et al., 2021; Kanyange et al., 2014). This demonstrates the clear potential for these communities to contribute to regulatory monitoring efforts, thereby reducing associated monitoring costs and improving spatial coverage.

In the present study, we show that trained local community citizen scientists can accurately monitor the concentrations of dissolved nutrients and particulates in the coastal waters of Lake Tanganyika. Nitrate measurements showed an accuracy of 91% (Cohen’s κ = 0.85) with a limited tendency for overestimation relative to the data produced by professional scientists. This limited overestimate may be related to the Griess method used by citizen scientists that include the concentration of nitrite within that measured for nitrate. While the ratio of nitrate to nitrate usually favours the former by orders of magnitude, modifications to the dissolved organic matter and oxygen concentrations can allow nitrite concentrations to become significant with respect to nitrate. Nearly all (98%) nitrate measurements reference measurements in the laboratory were either within the exact same or off by one concentration range category of the citizen scientist test kits. These values show that the distribution and accuracy of nitrate concentrations compared to professional values are in line with those reported by other citizen science programs that have analysed nitrate (Hadj-Hammou et al., 2017; McGoff et al., 2017; Scott & Frost, 2017). Importantly, the accuracy of citizen scientist nitrate measurements was not influenced by the increased precipitation and associated particulate loads typical of the wet season (with overall dry and wet accuracy of 89% and 91%, respectively). Phosphate measurements made by the citizen scientists had lower accuracy (74%; Cohen’s κ = 0.61) compared to accuracy of 81% reported by Lévesque et al. (2017); difference in trophic levels of two studied waterbodies can explain for this dissimilarity. However, nearly all (96%) of the measurements were either exact or within one category of the laboratory measurements. Similarly, there was a higher probability of overestimation of phosphate concentrations by citizen scientists. There was a clear reduction in accuracy during the wet season (66%) compared to the dry season (87%). These two observations (overestimates and reduced accuracy in the wet season) could be associated with either a lower accuracy of the phosphate method at higher concentrations or a possible influence of increased particulate concentrations. Similar findings were also reported by Muenich et al., 2016. Regarding the accuracy at higher concentrations, this seems unlikely as the accuracy in the highest category (0.10–0.20 mg/L P-PO_4_) was higher (75%) than the overall accuracy of all concentration categories. This points to the possible influence of particulate matter on phosphate estimates made by citizen scientists. It should be noted that samples obtained for laboratory analysis were filtered using a 0.45-micron glass fibre filter, while the sample tubes of the citizen scientists used unfiltered samples. Given the high affinity of phosphorus to adsorb on soil particles (Zhou et al., 2005), the overestimation by citizen scientists and the lower accuracy in conditions of high particulate matter during the wet season suggests that phosphate-loaded particulate matter may have increased phosphate concentrations detected by citizen scientists. The relative ratio of phosphate to TSS did not change between seasons, pointing to the increased concentrations of particulate matter, both TSS and turbidity, in the wet season.

Our results were comparable to similar studies using the same nutrient and particulate concentration methods (McGoff et al., 2017; Scott & Frost, 2017) and showed a higher accuracy than stand ard test strips (Muenich et al., 2016). It should be noted that colorimetric test methods have limitations due to assigning a colour impression to a concentration category, rather than a continuous scale and with the typical errors associated to judgement and colour interpretation (Burggraaff et al., 2021; Quinlivan et al., 2020; Storer et al., 2016).

In the same manner, citizen scientists were able to provide useful information on the concentration of suspended matter using low-cost turbidity tubes. Measuring turbidity using Secchi disks has been often used as a proxy of total suspended solids in lakes and marine waters (Davies‐Colley & Close, 1990; Hughes et al., 2015). Secchi tube allows for similar results in shallower coastal waters and rivers (Cunha et al., 2017; Miguel-Chinchilla et al., 2019).

### Detecting regional differences

The success of these methods suggests that they could be used to identify conditions where coastal water quality has been compromised, allowing for more focused mitigation actions. In the wet season, coastal lake conditions showed an elevated nutrient and particulate conditions, with higher total nitrogen and phosphorus, phosphates, nitrates, total suspended solids, and turbidity. There was a moderate-to-strong relationship between average monthly rainfall and nitrate concentrations (r = 0.61, n = 12), which was much higher than the low correlation of phosphate (r = 0.31, n = 12) (Cohen et al., 2005). Increased nutrient concentrations may result from atmospheric deposition (Gao et al., 2018) as well as run-off from agriculture and local village wastewater. Runoff from agricultural activities including animal keeping has been shown to provide nutrients inputs to Lake Tanganyika (Azanga, 2016). Likewise, wet atmospheric deposition has been shown to provide up to 83% of dissolved inorganic nitrogen, more than 30% of total phosphorus, 63% of dissolved phosphorus and 65% of soluble reactive phosphorus (Gao et al., 2018; Langenberg et al., 2003). Atmospheric deposition in other African Great lakes (Lake Victoria and Malawi/Nyasa/Niasa) has also been shown to be significant (Kishe, 2004; Tamatamah et al., 2005). However, nutrient conditions in coastal waters of nearshore villages are more likely to be impacted by local wastewater and agricultural land management (Kelly et al., 2017). This was evidenced by the differences between villages investigated in the present study and the importance of distance from the nearest river.

Among the five studied sites, Ilagala and Ujiji showed elevated nitrate, total nitrogen, total phosphorus, total suspended solids, and turbidity, while the lowest concentrations were found in Gombe (Table 3). Ilagala lies in the lake area influenced by the Malagarasi River, while Ujiji receives effluents from the much smaller Luiche-Ujiji River (Shen et al., 2022). Both rivers have been identified as important sources of particulates to Lake Tanganyika (Langenberg et al., 2003; Shen et al., 2022). Turbidity is expected to decrease exponentially with distance from the river mouth in deep lakes (Giovanoli, 1990). The catchments of rivers are characterised by a range of anthropogenic activities (Moshi et al., 2022). Conversely, Gombe is located in a protected area and is surrounded by forest with only limited agricultural activities. The protected forest acts both as a buffer to nutrient and sediment transport to the lake, and a blocker to more extensive activities in the surrounding catchment (Cózar et al., 2007; Msaky et al., 2005).

### Estimating water quality effects of environmental change

Climate plays a major role in the circulation and dynamics of the African Great Lakes, and Lake Tanganyika, with its north–south extension of nearly 700 km, is highly sensitive to changing temperature and wind regimes (Kraemer et al., 2015; Loiselle et al., 2014; Mziray et al., 2018). Given the expected changes in the coming decades, combined with expected increase in the population of coastal areas, a logistic model, based on monthly measurements in 15 sites, indicates that the nutrient conditions in the coastal waters of the lake are expected to worsen, in particular in the wet season and regarding total phosphorus and nitrogen. Particulate conditions are expected to show an increase in both seasons, where the probability of TSS remaining above national limits continuing throughout the year is vivid. It should be noted that the climate scenario applied (A2) was at the high end of the emission scenarios considered in the IPCC's Special Report on Emissions Scenarios (Nakicenovic & Swart, 2000).

The expected probabilities of increasing nutrient and particulate conditions result from expected changes in precipitation, population and wind regimes. Wind intensity and direction effects both atmospheric nutrient transport and vertical and horizontal mixing of dissolved and particulate matter in lakes (Mziray et al., 2018).

Increasing wind speed will likely decrease the concentration of phosphate, total nitrogen, and total phosphorus. Wind speed has an important role in transporting and activating the sediment layer to trigger particles entrainment into the overlying water and release nutrients. The same explanation was given by Deng et al. (2018) and Tang et al. (2020), who observed that the eutrophication of Lake Taihu where wind speed played a significance role of releasing particulate nutrients resulting into increased concentrations of total nitrogen, total phosphorus and total suspended solids.

Population was significant in the modelling of total nitrogen concentration probabilities. Kigoma is among the fast-growing towns in Tanzania, and due to increase in human population (NBS, 2013), forest has been cleared out for settlement and farming activities. Increases in the use of artificial fertilizers are likely to increase nutrient transport to the lake basin easily. Rapid increase in population and economic activities has driven increases in nitrogen in other large lakes (Chen et al., 2022; Gao et al., 2015).

### Conclusion

We demonstrated that citizen scientists produce reliable water quality data in the complex coastal conditions of Lake Tanganyika. Their measurements indicate that water quality is strongly influenced by climate conditions and local factors, creating conditions where many national and international guidelines on nutrient and particulate concentrations are surpassed for most of the year, particularly in the rainy season. Extrapolating these relationships to expected changes in climate (precipitation) and population, there is a clear risk of worsening conditions in the coming decades. This calls for increased local and transnational efforts to better regulate land -use activities such as agriculture, improve wastewater management and engage coastal communities to reduce nutrients and particulate matter loads to the lake, especially with respect to nutrient and sediment input pathways that are associated with rainfall. To achieve such goals, upscaling citizen science to more communities around Lake Tanganyika, and to other African Great lakes, would increase awareness of environmental problems and could bring together citizens, regulators, research institutions, and Non-Governmental Organizations (NGOs) in order to conserve the lake ecosystem services, inform management and mitigation actions, and support long-term decision-making.

## Data Availability

The datasets used in present study are available on the FreshWater Watch online database (https://freshwaterwatch.thewaterhub.org/our-data/explore-our-data) for all citizen science data (past and present) and by request from the corresponding authors for the laboratory data used for validation.
